# Understanding factors influencing the length of hospital stay among non-severe COVID-19 patients: A retrospective cohort study in a Fangcang shelter hospital

**DOI:** 10.1371/journal.pone.0240959

**Published:** 2020-10-21

**Authors:** Shishi Wu, Lanping Xue, Helena Legido-Quigley, Mishal Khan, Hua Wu, Xiaoxiang Peng, Xuewen Li, Ping Li

**Affiliations:** 1 Saw Swee Hock School of Public Health, National University of Singapore, Singapore, Singapore; 2 Department of Neurology, Shanxi Bethune Hospital, Shanxi Academy of Medical Science, Taiyuan, Shanxi, China; 3 Faculty of Public Health and Policy, London School of Hygiene & Tropical Medicine, London, United Kingdom; 4 Department of Orthopedics, Shanxi Bethune Hospital, Shanxi Academy of Medical Science, Taiyuan, Shanxi, China; 5 Department of Neurology, The Third People’s Hospital of Hubei Province, Wuhan, Hubei, China; 6 Department of Cardiovascular Medicine, Shanxi Bethune Hospital, Shanxi Academy of Medical Science, Taiyuan, Shanxi, China; 7 Department of Respiratory and Critical Care Medicine, Shanxi Bethune Hospital, Shanxi Academy of Medical Science, Taiyuan, Shanxi, China; University Magna Graecia of Catanzaro, ITALY

## Abstract

As a novel concept of responding to disease epidemics, Fangcang shelter hospitals were deployed to expand the health system’s capacity and provide medical services for non-severe COVID-19 patients during the outbreak in Wuhan. To give insights on patient management within Fangcang hospitals, we conducted a retrospective analysis to: 1) describe the characteristics of the patients admitted to Fangcang hospitals and 2) explore risk factors for longer length of stay (LOS). We enrolled 136 confirmed COVID-19 patients, including asymptomatic patients and those with mild symptoms, who were hospitalized in the Wuti Fangcang Hospital. 58 patients completed the treatment and discharged before 1 March 2020. After describing patients’ demographic and clinical characteristics, exposure history, treatment received and time course of the disease, we conducted linear regression analysis to identify factors influencing LOS. We found that patients having fever before admission were hospitalized 3.5 days (95%CI 1.39 to 5.63, p = 0.002) longer than those without fever and that patients having bilateral pneumonia were hospitalized 3.4 days (95%CI 0.49 to 6.25, p = 0.023) longer than those with normal CT scan results. We also found weak evidence suggesting that patients with diabetes were hospitalized 3.2 days longer than those without diabetes (95%CI -0.2 to 6.56, p = 0.065). However, we observed no significant differences in LOS between symptomatic and asymptomatic patients and between patients who received treatment and those without treatment. Longer duration of hospitalization among non-severe COVID-19 patients is associated with having fever, bilateral pneumonia on CT scan and diabetes. However, being asymptomatic and using supportive medications at the early stage of infection do not have significant influences on LOS. Our study is a single-centered study with relatively small sample size. The findings provide evidence for predicting hospital bed demand in a novel response scenario and may help decision-makers in preparing for ramping up the health system capacity.

## Introduction

COVID-19, caused by the severe acute respiratory syndrome coronavirus 2 (SARS-CoV-2), has rapidly become a global pandemic since it was first reported in December 2020. As of 8 July 2020, over 11 million cases and 535,759 deaths have been reported worldwide [1]. The COVID-19 pandemic not only overburdens the intensive care units with influx of critically ill patients, but also challenges the health systems’ capacity to respond to the needs of non-severe patients who require necessary examinations and treatment.

To accommodate for the surge of COVID-19 patients in Wuhan—the former epicenter of China–in January and February 2020, the Chinese government successfully expanded the health system’s capacity in a short time by implementing a patient triage scheme and adopting a novel concept of responding to public health emergency–Fangcang hospitals. Converted from stadium or exhibition centers, Fangcang hospitals are temporary hospitals providing medical care during large-scale emergencies, such as earthquakes or floods. According to the COVID-19 diagnosis and treatment guideline (6th edition) in China, confirmed COVID-19 cases were categorized into four groups—minor, moderate, severe and critical—based on clinical manifestations [2]. Patients with minor and moderate symptoms were admitted to Fangcang hospitals once they were diagnosed with COVID-19. Additionally, since frontline doctors reported cases of asymptomatic and presymptomatic transmission, asymptomatic patients identified from quarantine sites or screening of close contacts of confirmed cases were also admitted to Fangcang hospitals [3–5]. If patients’ conditions deteriorated, they would be transferred to one of the designated tertiary hospitals where critical care was available. In a published article, the authors highlighted the functions of Fangcang hospitals and emphasized their critical roles in the national responses to the COVID-19 epidemics in China [6]. However, studies giving more insights on patient management within Fangcang hospitals and the characteristics of patients in these hospitals are scarce [7].

Furthermore, although strict social confinement measures seem to be effective in reducing case incidence and mortality in the first wave [8–11], as many countries have already lifted lockdown or are in the process of doing so, there is a great risk of a larger second wave of COVID-19 happening in the following months, which poses a major threat to the already taxed health systems in many countries [12, 13]. Therefore, early preparation to ramp up the health system capacity is crucial in the event of the second wave [13, 14].

Understanding how long COVID-19 patients require healthcare in hospitals is important for predicting hospital bed demand and planning resource allocation, particularly in resource constraint settings. Many existing studies suggest that patients’ characteristics that influence disease severity are likely to influence the length of stay (LOS) in hospitals [15–19], but no conclusion is drawn because LOS is often reported as one of the secondary outcomes without being further analyzed [20]. We only found three studies that investigated the risk factors influencing LOS among COVID-19 patients [21–23]. In these studies, prolonged hospitalization of COVID-19 patients was found to be associated with being female, having fever, having chronic liver or kidney disease on admission, higher creatine level, lymphopenia, and bilateral pneumonia on CT scan [21, 22]. However, no consensus on particular risk factors has been reached.

Therefore, the purpose of this study is to: 1) describe the characteristics of the non-severe patients who were hospitalized in Fangcang hospitals; 2) explore risk factors for longer LOS among non-severe COVID-19 patients using the clinical data collected from a Fangcang hospital in Wuhan.

## Materials and methods

### Study setting

As one of the 16 Fangcang hospitals operated during the COVID-19 outbreak in Wuhan, the Qiaokou district Wuti Fangcang Hospital (Qiaokou Fangcang Hospital) started to receive patients on 11 February 2020. A medical team from Shanxi province was responsible for providing health services at the Qiaokou Fangcang Hospital, with assistance from the local government and a local tertiary hospital. Patients who were admitted to Fangcang hospitals met the following criteria: 1) tested positive in RT-PCR test for SARS-CoV-2; 2) having self-care ability; 3) having mild or moderate symptoms; 4) no severe comorbidities (such as heart failure, renal failure) or mental disorders; 5) tested negative in an influenza virus RT-PCR test. Asymptomatic patients identified from quarantine sites or screening of the close contacts of confirmed cases were also admitted to Fangcang hospitals for isolation.

Apart from its function of isolating and triaging COVID-19 patients, the Qiaokou Fangcang Hospital provided the following essential services. First, it served as a shelter that provided essential living necessities to patients, such as accommodation, food, sanitation services and social engagement. Second, patients’ conditions were monitored by the healthcare providers, as body temperature, blood pressure and oxygen saturation were checked multiple times daily. Mobile CT scanner and blood testing lab were available onsite for more specialized examinations. Third, basic medical care, including oral and intravenous medications, oxygen supplementation and mental health counseling, were available and provided to those patients in need.

### Study participants

Patients meeting the following criteria were discharged: 1) having a normal body temperature for three consecutive days; 2) no significant respiratory symptoms; 3) significant improvement of pneumonia on chest CT scan; 4) two consecutive negative results for RT-PCR test 24 hours apart.

On the contrary, if patients’ condition deteriorated and met one of the following criteria, the patients were transferred to a designated hospital for further treatment: 1) respiratory rate ≥ 30/minute; 2) blood oxygen saturation ≤93% on room air; 3) oxygenation index ≤ 300 mmHg; 4) body temperature ≥ 38.5 Celsius degree for two consecutive days after treatment; 5) comorbidities deteriorated severely during hospitalization; 6) other emergency medical reasons.

**Fig 1:**
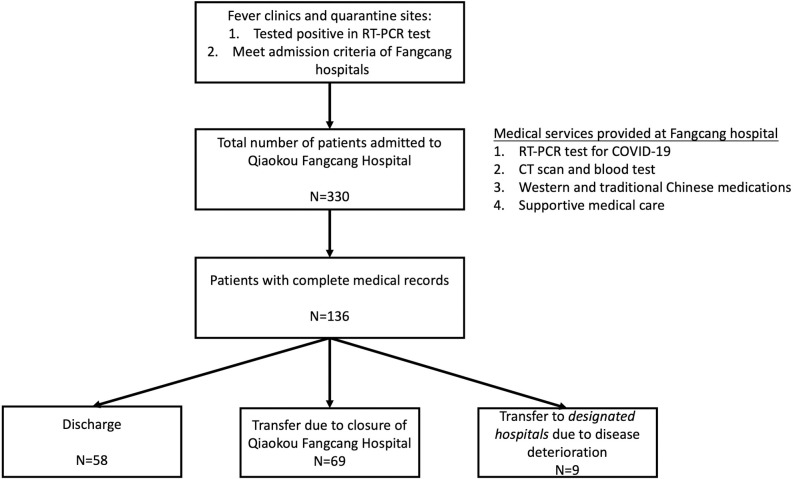
Flow chart of patients admitted to the Qiaokou Fangcang Hospital.

Fig 1 shows the triage flow at the Qiaokou Fangcang Hospital. A total of 330 confirmed COVID-19 patients were admitted to the Qiaokou Fangcang Hospital between 11 February and 1 March 2020. To improve the efficiency of patient management, an electronic medical records system was developed onsite. After the launch of the electronic system, health information of 136 patients who were admitted to the Qiaokou Fangcang Hospital was updated to and stored in the system. Among the 136 patients with complete medical records, while nine patients were transferred to a designated hospital due to disease deterioration, 58 patients met the criteria and were discharged before 1 March 2020. Because the Qiaokou Fangcang hospital was closed on 1 March 2020, patients with mild symptoms who did not meet the discharge criteria were transferred to another hospital for continuing isolation and treatment. In this study, we only included the 58 patients who recovered and were discharged before the closure of the Qiaokou Fangcang hospital.

### Data collection

Patients’ health information was collected between 25 February and 1 March 2020 by the medical team from the Shanxi Bethune Hospital and stored in the electronic medical records system. In addition to the date of hospital admission and discharge, the following data was extracted from the electronic system by two researchers (LP and XLP): 1) patients’ demographic information (age and gender); 2) clinical information, including comorbidities, smoking status, levels of white blood cell (WBC), lymphocyte and c-reactive protein (CRP), CT scan results; 3) exposure history, including contact mode, number of residents in the household and contact length; 4) symptoms and the date of symptom onset; 5) treatment received before admission to the Fangcang Hospital. Raw data was compiled in an excel sheet, which was imported to and analyzed using STATA V.14.1.

### Statistical analysis

The primary outcome of this study is the LOS at the Fangcang Hospital, which was calculated as the number of days that patients were hospitalized from admission to discharge.

We first conducted a descriptive analysis of patients’ demographic and clinical characteristics, exposure history, common symptoms, treatment received and time course of the disease. The distribution of continuous variables was assessed using the Shapiro-Wilk test. For normally distributed variables, means with standard deviation were calculated, while medians and IQR were used to describe variables not normally distributed. Categorical variables were described as frequency and percentage. To assess the differences in the demographic and clinical characteristics between symptomatic and asymptomatic patients, we compared the means and medians of continuous variables using the student’s t-test and Mann-Whitney U test respectively. The differences in percentages of categorical variables between the two groups were assessed using the chi-square test.

To identify factors influencing the LOS of patients at the Fangcang hospital, we conducted multivariable linear regression analysis. The association between the outcome variable (LOS) and each covariate was first assessed in univariable analysis. We performed a backward stepwise selection to identify covariates to be included in the final multivariable linear regression model. The selection was based on the Akaike Information Criterion (AIC): the added variable would be included if the model gave a smaller AIC value compared with the one without the added variable, suggesting that the model has better fitness with less complexity [24]. The regression coefficient of each included covariate, the associated 95% confidence interval (CI) and the two-sided p-value were reported. For all the statistical tests, a p-value less than 0.05 was considered statistically significant.

### Ethical approval

Approval was taken from the Qiaokou Fangcang Hospital and the ethics committee of the Shanxi Bethune Hospital (YXLL-KY-2020-001). Written informed consent was waived given the context of a public health emergency.

## Results

### Patients’ characteristics and exposure history

In this study, we included a total of 58 patients who were hospitalized in the Qiaokou Fangcang Hospital and discharged between February 11th and March 1st, 2020. Among these patients, 13 (22.4%) were identified as asymptomatic on admission. As shown in Table 1, the median age of the patients was 55.5 years old (IQR = 20), and about 62.1% (N = 36) were female. We also compared the demographic characteristics between the symptomatic and asymptomatic patients. We observed that the asymptomatic group had an average age (mean = 52, SD = 14) less than the symptomatic group (mean = 57, SD = 20) by 5 years and a higher proportion of females (N = 10, 76.9%) than the symptomatic group (N = 26, 57.8). However, the differences in age and gender between the two groups were not significant.

**Table 1:** Demographic and clinical characteristics of the patients and their exposure history.

	Total (N = 58)	Severity		
		Symptomatic (N = 45)	Asymptomatic (N = 13)	p-value
**Demographic characteristics**				
Age, median (IQR)	55.5 (20)	57 (20)	52 (14)	0.28
Gender, N(%)				0.21
Male	22 (37.9)	19 (42.2)	3 (23.1)	-
Female	36 (62.1)	26 (57.8)	10 (76.9)	-
**Exposure history**				
**Contact mode, N (%)**				0.056
Been to Huanan see market	1 (1.7)	1 (2.2)	0 (0)	-
Contact with confirmed family member(s)	32 (55.2)	22 (48.9)	10 (76.9)	-
Contact with confirmed friends or colleagues	3 (5.2)	1 (2.2)	2 (15.4)	-
Involved in mass gathering	4 (6.9)	4 (8.9)	0 (0)	-
Don’t know	18 (31)	17 (37.8)	1 (7.7)	-
**Number of people in the household, median (IQR)**	3 (2)	3 (2)	3 (3)	0.33
**Contact length, N (%)**				0.088
Less than 1 hr	2 (3.5)	1 (2.2)	1 (7.7)	-
Longer than 1 hr and less than 24 hrs	6 (10.3)	5 (11.1)	1 (7.7)	-
Longer than 24 hours	30 (51.7)	20 (44.4)	10 (76.9)	-
Don’t know	20 (34.5)	19 (42.2)	1 (7.7)	-
**Clinical characteristics**				
**Any comorbidity, N (%)**	22 (37.9)	19 (42.2)	3 (23.1)	0.21
Hypertension	13 (22.4)	11 (24.4)	2 (15.4)	0.5
Cardiovascular disease	3 (5.2)	3 (6.7)	0 (0)	0.34
Diabetes	6 (10.3)	6 (13.3)	0 (0)	0.16
Chronic liver disease	1 (1.7)	1 (2.22)	0 (0)	0.59
**Smoking**	6 (10.3)	5 (11.1)	1 (7.7)	0.72
**WBC (x 10**^**9**^**)**				0.374
≥4 and ≤10	35 (60.3)	29 (64.4)	6 (46.1)	
<4	1 (1.72)	1 (2.2)	0 (0)	
>10	22 (37.9)	15 (33.3)	7 (53.9)	
**Lymphocyte (%)**				0.463
≥20 and ≤40	27 (46.5)	22 (48.9)	5 (38.5)	
<20	8 (13.8)	7 (15.5)	1 (7.7)	
>40	23 (39.7)	16 (35.6)	7 (53.8)	
**CRP (mg/L)**				0.052
≤10	17 (29.3)	16 (35.6)	12 (92.3)	
>10	41 (70.7)	29 (64.4)	1 (7.7)	
**Chest CT scan**				<0.001
None	10 (17.2)	3 (6.7)	7 (53.9)	-
Unilateral pneumonia	12 (20.7)	9 (20.0)	3 (23.1)	-
Bilateral pneumonia	36 (62.1)	33 (73.3)	3 (23.1)	-
**Outcome**				
Length of stay (LOS), mean (SD)	10.3 (4.9)	10.8 (5.2)	8.4 (3.4)	0.12

Apart from the 18 patients (31%) who did not recall their exposure history and 1 (1.7%) who had visited the Huanan sea market, most of the patients (N = 32, 55.2%) were close contacts of their confirmed family members, with fewer patients being exposed to confirmed friends/colleagues (N = 3, 5.2%) or at mass gatherings (N = 4, 6.9%). The median number of residents in a household was three (IQR = 2), and most of the patients (N = 30, 51.7%) had contact with confirmed cases for longer than 24 hours. We also observed that 76.9% (N = 10) of the asymptomatic patients had contact with confirmed family members, which is higher than the proportion among symptomatic patients (N = 22, 48.9) by 28% (p = 0.056).

The clinical characteristics of the study participants are also presented in Table 1. Hypertension (N = 13, 22.4%) and diabetes (N = 6, 10.3%) were the most common comorbidities reported by the patients. Only six participants (10.3%) reported a history of smoking. The asymptomatic group had a lower proportion of patients with comorbidities (N = 3, 23.1%) than the symptomatic group (N = 19, 42.2%), but the difference was not significant (p = 0.21). While 37.9% (N = 22) and 39.7% (N = 23) of the patients had increased WBC level and lymphocyte level respectively, most of the patients had normal WBC (N = 35, 60.3%) and lymphocyte (N = 27, 46.5%) levels. No significant difference in WBC and lymphocyte levels between symptomatic and asymptomatic groups was observed. At the time of hospital admission, most of the patients (N = 41, 70.7%) had an elevated CRP level. We observed that about 7.7 (N = 1) of the asymptomatic patients had a CRP level over 10mg/L, which is significantly lower than the proportion among symptomatic patients (N = 29, 64.4%) by 27.9% (p = 0.052). The CT scan results showed that among symptomatic patients, 73.3% (N = 33) developed bilateral pneumonia and only 3 (6.7%) patients had normal results. Three asymptomatic patients (23.1%) developed unilateral pneumonia and three (23.1%) developed bilateral pneumonia, even though they reported no perceivable symptoms at the time of hospital admission. The proportion of bilateral pneumonia among the symptomatic group was significantly higher than the proportion of the asymmetric group by 50.2% (p<0.001).

The mean time for hospitalization at the Fangcang hospital, which is the primary outcome in our study, was 10.3 days (SD = 4.9). Even though asymptomatic patients (8.4 days, SD = 3.4) had a shorter LOS than symptomatic patients (10.8 days, SD = 5.2) by 2.4 days, the difference was not significant (p = 0.12).

### Common symptoms

As reported by the symptomatic patients (N = 45), cough (N = 24, 41.4%) was the most common symptom at onset of illness, followed by fever (N = 23, 39.7) and fatigue (N = 21, 36.2%). Less common symptoms were headache (N = 12, 20.7%), diarrhea (N = 6, 10.3%) and vomit (N = 5, 8,6%) (Table 2).

**Table 2:** Early symptoms among symptomatic patients.

Symptoms	Number of patients (%)
Cough	24 (41.4)
Phlegm	13 (22.4)
Fatigue	21 (36.2)
Myalgia	13 (22.4)
Headache	12 (20.7)
Dyspnea	13 (22.4)
Fever	23 (39.7)
Diarrhea	6 (10.3)
Vomit	5 (8.6)

### Treatment received before admission and time course of COVID-19 among study participants

As shown in Table 3, most patients (N = 41, 70.7%) had taken medications at the early stage of infection before admitted to the Fangcang Hospital: almost half of the patients (N = 28, 48.3%) received a combination of TCM and western medications (antiviral drugs or antibiotics); three patients (5.2%) were prescribed with only western medications (antiviral drugs or antibiotics); ten patients (17.2%) took TCM only. As reported by the patients, Oseltamivir and Arbidol were the most commonly prescribed antiviral drugs. The antibiotics that the patients had received included Moxifloxacin, Levofloxacin and Erythromycin.

**Table 3:** Treatment received before admission to the Qiaokou Fangcang Hospital and the time course of COVID-19 among study participants.

Treatment received before admission to Fangcang hospital	Number of patients (%)
None	17 (29.3)
TCM and antiviral or antibiotics treatments	28 (48.3)
Antiviral or antibiotics treatments	3 (5.2)
TCM only	10 (17.2)
**Time course**	
Duration from symptom onset to admission to Fangcang (days), median (IQR)	13 (20)
Duration from symptom onset to discharge (days), median (IQR)	25 (20)

For all the patients, the median durations from symptom onset to admission to the Fangcang Hospital and from symptom onset to discharge were 13 days (IQR = 20) and 25 (IQR = 20) days respectively.

### Factors associated with length of hospitalization at the Fangcang Hospital

Results from the linear regression analysis in which we explored factors associated with the length of hospital stay at the Qiaokou Fangcang Hospital are shown in Table 4. In the univariable analysis, we found that LOS was significantly associated with gender, having comorbidities (cardiovascular disease and diabetes), lymphocyte level, CT scan results, fever and duration from symptom onset to hospital admission. Even though we observed a shorter LOS in the groups of patients who were treated with only TCM (coefficient = -1.02, 95%CI -7.29 to -5.25, p = 0.746) and only western medications (coefficient = -1.85, 95%CI -5.84 to 2.14, p = 0.356) than in the non-treatment group, the differences were not significant.

**Table 4:** Factors associated with LOS.

	Univariable analysis		Multivariable analysis	
	Coefficient (95% CI)	p-value	Coefficient (95% CI)	p-value
**Asymptomatic**				
No	Reference	-	-	-
Yes	-2.44 (-5.52, 0.64)	0.119	-	-
**Age**	0.08 (-0.01, 0.17)	0.096	-	-
**Gender**				
Male	Reference	-	-	-
Female	-2.63 (-5.24, -0.02)	0.048	-	-
**Any comorbidity**	1.75 (-0.91, 4.42)	0.193	-	-
Hypertension	2.02 (-1.08, 5.13)	0.197	-	-
Heart disease	-6.3 (-11.95, -0.58)	0.031	-	-
Diabetes	5.64 (1.60, 9.68)	0.007	3.18 (-0.20, 6.56)	0.065
Chronic liver disease	-1.29 (-11.38, 8.78)	0.797	-	-
Smoking	1.92 (-2.36, 6.20)	0.372	-	-
**Number of people in the household, median**	0.56 (-0.18, 1.30)	0.138	-	-
**Contact length**				
Less than 1 hr	Reference	-	-	-
Longer than 1 hr and less than 24 hrs	4.5 (-3.73, 12.73)	0.278	-	-
Longer than 24 hours	3.27 (-4.09, 10.63)	0.378	-	-
Don’t know	3.25 (-4.23, 10.73)	0.387	-	-
**WBC (x 10**^**9**^**)**				
≥4 and ≤10	Reference	-	-	-
<4	-6.31 (-16.40, 3.77)	0.215	-	-
>10	0.19 (-2.52, 2.89)	0.891	-	-
**Lymphocyte (%)**				
≥20 and ≤40	Reference	-	-	-
<20	4.8 (0.95, 8.7)	0.015	-	-
>40	1.36 (-1.35,4.07)	0.319	-	-
**CRP (mg/L)**				
≤10	Reference	-	-	-
>10	-1.61 (-4.46, 1.25)	0.254	-	-
**Chest CT scan**				
None	Reference	-	Reference	-
Unilateral pneumonia	-0.3 (-4.22, 3.62)	0.879	-0.28 (-3.48, 2.92)	0.861
Bilateral pneumonia	4.09 (0.82, 7.36)	0.015	3.37 (0.49, 6.25)	0.023
**Cough**	1.16 (-1.48, 3.81)	0.382	-	-
**Phlegm**	1.73 (-1.39, 4.84)	0.272	-	-
**Fatigue**	1.88 (-0.80, 4.57)	0.166	-	-
**Muscle ache**	-0.95 (-4.09, 2.19)	0.547	-	-
**Headache**	-0.45 (-3.69, 2.79)	0.780	-	-
**Heard to breathe**	0.83 (-2.31, 3.98)	0.597	-	-
**Fever**	3.22 (0.68, 5.76)	0.014	3.51 (1.39, 5.63)	0.002
**Diarrhea**	1.55 (-2.73, 5.84)	0.472	-	-
**Vomit**	0.79 (-3.88, 5.47)	0.735	-	-
**Interval between symptom onset to admission to Fangcang (days)**	-0.16 (-0.27, -0.05)	0.005	-0.21 (-0.30, -0.12)	<0.001
**Treatment received before Fangcang**				
None	Reference	-	-	-
TCM and antiviral or antibiotics treatments	0.61 (-2.47, 3.69)	0.692	-	-
Antiviral or antibiotics treatments	-1.02 (-7.29, 5.25)	0.746	-	-
TCM only	-1.85 (-5.84, 2.14)	0.356	-	-

In the multiple variable linear regression analysis, we included the following variables in our final model: diabetes, durations from symptom onset to admission, fever and chest CT scan results. Our analysis showed that longer LOS was significantly associated with having fever on admission, shorter duration from symptom onset to admission, and having bilateral pneumonia after adjusting for other covariates. We found strong evidence to indicate that patients having fever before admission had significantly longer LOS than those without fever by 3.5 days (95%CI 1.39 to 5.63, p = 0.002) and that patients having bilateral pneumonia were hospitalized 3.4 days (95%CI 0.49 to 6.25, p = 0.023) longer than those with normal CT scan results. We also observed that patients who experienced shorter duration from symptom onset to admission had significantly longer LOS. Even though the evidence was strong (p<0.001), the magnitude of this association was very small (coefficient = -0.21, 95%CI -0.30 to -0.12). Furthermore, we found weak evidence showing an association between LOS and diabetes after adjusting for other variables. Patients with diabetes were hospitalized 3.2 days longer than those without diabetes (95%CI -0.2 to 6.56, p = 0.065).

## Discussion

In this retrospective cohort study of non-severe COVID-19 patients admitted to a Fangcang hospital, we found that longer hospitalization is associated with having fever, bilateral pneumonia, shorter duration from symptom onset to admission and diabetes. However, there is no significant difference in the LOS at Fangcang hospitals between asymptomatic and symptomatic patients and between patients who received medications and those without treatment before hospital admission.

According to a systematic review of COVID-19 length of hospital stay, the median LOS at general hospitals (non-ICU) in China is 14 days (IQR:10–19) [20], which is four days longer than the median in our study. This can be explained by the different admission criteria between Fangcang hospitals and general hospitals outside of Wuhan. In Wuhan, patients were screened for disease severity and only non-severe patients (asymptomatic patients and those with minor and moderate symptoms) were referred to Fangcang hospitals. This patient triage scheme was only implemented in Wuhan to accommodate the rapid surge of cases in January and February 2020, but not in the other cities because the outbreak was soon contained after the lockdown of Wuhan. As a result, patients outside of Wuhan were admitted to and hospitalized in local general hospitals regardless of disease severity. In many existing studies conducted outside of Wuhan, both non-severe and severe patients who were hospitalized in tertiary hospitals were included in the study populations [15, 22, 25, 26]. However, studies in the setting of Fangcang hospitals were scarce. To our knowledge, only one study focused on the clinical characteristics of patients stayed in a Fangcang hospital in Wuhan; however, it did not report LOS as an outcome indicator, thus a comparison was not possible [7].

We found that the duration of hospital stay is associated with two signs of the inflammatory response—fever and bilateral pneumonia on chest CT scan–among non-severe COVID-19 patients. Fever has been used widely as an indicator for screening people suspected of COVID-19 and assessing disease severity in the COVID-19 diagnostic and treatment guidelines in China [2, 27–29]. It is the most reported symptom in COVID-19 patients and found to be more common in severe patients, as a meta-analysis found that the incidences of fever in the total study population and in the severe patients were 89.1% and 93.5% respectively [30]. Despite that fever was less common in our study than in the other studies due to the admission criteria of Fangcang hospitals that excluded severe COVID-19 cases, our findings suggest that patients who reported having fever at the early stage of infection require longer hospitalization, which is consistent with previous studies [21, 22]. Similarly, as a result of pulmonary inflammation caused by viral infection, radiological changes in the lungs is common among COVID-19 patients. As reported in a meta-analysis of 43 studies, 73% of patients developed bilateral pneumonia [29]. We observed a similar prevalence of bilateral pneumonia and confirmed with a previous study that bilateral pneumonia on CT scan is associated with longer duration of hospital stay [21].

Asymptomatic patients have less severe clinical manifestations than symptomatic patients, as the prevalence of bilateral pneumonia was significantly lower than the prevalence in the symptomatic group. However, we observed no significant difference in LOS between the two groups. This could be explained by that asymptomatic and symptomatic patients have similar viral loads, as reported in a previous study [31]. It is also possible that some patients were in presymptomatic phase when they were confirmed with COVID-19. Additionally, despite that a study of 24 asymptomatic cases suggest that young patients less than 15 years old are more likely to be asymptomatic [32], we did not find any significant differences in demographic characteristics or the prevalence of comorbidities between asymptomatic and symptomatic patients due to older age group of our study participants and small sample size.

Diabetes is the second most prevalent comorbidity among COVID-19 patients [33]. The prevalence of diabetes in our study is similar to its prevalence among other COVID-19 study populations (9.7%) and the total population (10.9%) in China [33]. Although it is still inconclusive whether diabetes is a risk factor for severe COVID-19 [33, 34], we found weak evidence suggesting that diabetes is associated with longer hospital stay among non-severe COVID-19 patients. One explanation could be that diabetes may impair the functions of macrophage and lymphocyte and adversely affect T-cell growth and production of interferon γ, thus leading to suppressed immunological function [35, 36]. It is also possible that viral infection, such as influenza, may lead to fluctuations in blood glucose level, which may exacerbate the complications of diabetes and prolong the recovery process [37–39].

Additionally, we observed that pre-existing cardiovascular comorbidities, such as hypertension and heart diseases, were prevalent among our participants. Although we did not observe significant associations between cardiovascular diseases and LOS, previous studies have shown that they are risk factors for COVID-19 infection and poor prognosis [40, 41]. One explanation could be that the virus is able to bind to the angiotensin-converting enzyme 2 (ACE2), thus affecting the ACE2 signaling pathways and leading to acute cardiac injury [40]. Experts also suspect that the acute systemic inflammatory response caused by the uncontrolled release of pro-inflammatory cytokines may affect the cardiovascular system [40], as pro-inflammatory cytokines storm was observed among severe COVID-19 patients in several studies [27, 42, 43].

Consistent with other studies in China, antibiotics and antiviral medications are commonly prescribed to COVID-19 patients [26, 44, 45]. Although no proven effective antiviral treatment specifically for COVID-19 exists, antiviral medications such as Arbidol and Lopinavir are recommended in the early editions of COVID-19 diagnostic and treatment guidelines in China and drugs to relieve symptoms and inflammation responses are often prescribed by doctors at fever clinics [2]. Additionally, TCM has been used for treating viral infections, such as H1N1 and MERS, and recommended in the COVID-19 diagnostic and treatment guidelines in China [2, 46]. Although we did not find a significant difference in hospitalization duration between patients who received medications and those without pharmaceutical treatment, a number of medications are shown to be effective as supportive treatment of COVID-19. For example, early use of “Lianhuaqingwen” capsules–a Chinese proprietary medicine commonly used for treating cold and flu—has significantly improved the recovery of symptoms [47]. However, to reach a consensus on effective therapies, more clinical data on pharmaceutical treatment for COVID-19 are urgently needed.

### Strengths and limitations

Our study provides evidence for predicting hospital bed demand in a novel public health response scenario by examining the length of hospitalization—an important but underreported indicator—and its associated factors among non-severe COVID-19 patients hospitalized in Fangcang hospitals. The findings are useful for decision-makers to prepare for ramping up the health system capacity and planning for resource allocation. In this study, we also give more insights on patient management within Fangcang hospitals and the characteristics of patients in these hospitals. Therefore, our study is informative for frontline healthcare providers in countries which are planning to adopt the “Fangcang” model. Finally, we highlight the influence of being asymptomatic and diabetic on LOS, which can be useful for clinicians when making treatment plans for those groups of COVID-19 patients.

Our study has the following limitations. First, this is a single-centered study with a relatively small sample size, which may limit our interpretation of the analysis results. Second, information on medical history, treatment received before admission to Fangfang hospitals and the date of diagnosis and symptom onset was reported by the patients and susceptible to recall bias. However, because of the retrospective cohort study design, errors in reporting are likely to be non-differential across the study participants. Finally, because a standard treatment was provided to all the patients during their hospitalization in Fangcang hospitals, we only included treatment received before admission to Fangcang hospitals as a variable in the analysis.

## Conclusion

In this retrospective cohort study of non-severe COVID-19 patients who were staying in a Fangcang hospital, we found that longer duration of hospitalization is associated with having fever, bilateral pneumonia on CT scan, shorter duration from symptom onset to admission and diabetes. However, being asymptomatic and using supportive medications at the early stage of infection do not have significant influences on the length of hospital stay. Our study provides evidence for predicting hospital bed demand in a novel public health response scenario where emergency hospitals are deployed to meet the needs of a large number of non-severe patients and may help decision-makers in the early preparation for the second wave of COVID-19 cases.
